# An in-home engagement and usability study of GeRI: an open-source platform for remote symptom assessment and wearable activity monitoring in men with prostate cancer

**DOI:** 10.3389/fdgth.2026.1700852

**Published:** 2026-03-20

**Authors:** Nabiel Mir, Yan Che, Mohammad Taha Bin Firoz, Mohammad Ziad Siddiqui, Megan Mendez, Megan Huisingh-Scheetz, Russell Z. Szmulewitz

**Affiliations:** 1 Department of Medicine, Section of Hematology/Oncology, The University of Chicago, Chicago, IL, United States; 2 Department of Public Health Sciences, The University of Chicago, Chicago, IL, United States; 3 Prosilient Systems Inc., Newark, DE, United States; 4 Department of Medicine, Section of Geriatrics and Palliative Medicine, The University of Chicago, Chicago, IL, United States

**Keywords:** decentralized clinical trials, digital biomarkers, frailty, open-source software, remote geriatric assessment, wearable sensors

## Abstract

Geriatric assessment (GA) is underused in oncology because clinic-based implementation is time- and resource-intensive, limiting routine evaluation of frailty and treatment tolerance. Existing digital tools often rely on proprietary devices and closed analytic pipelines. We developed the Geriatric Remote Initiative (GeRI), an open-source platform integrating a wrist-worn accelerometer, smart scale, and tablet interface with reproducible analytics and triggerable surveys. GeRI was evaluated in a 12-week home deployment among 10 men aged ≥65 years receiving androgen deprivation therapy for prostate cancer. Self-rated health and symptom surveys triggered 48-h accelerometry windows, initiated by pressing “Start monitoring” on the watch. Engagement was defined *a priori* as completion of ≥50% of prompted interactions (surveys, scale readings, and watch session initiation) within each 4-week block; prespecified engagement was met by 9/10 participants in all three blocks. Usability was assessed using the System Usability Scale (range 57.5–100; 8/10 ≥ 75). Across the first three 4-week blocks, survey and scale completion was high (86%–87% and 83%) while watch session initiation occurred in 65% of prompts. Initiated windows could contribute up to 80 recoverable 24-h intervals; 43 met validity criteria (≤1.5 h non-wear and ≥300 steps/day), and one participant contributed 0 valid intervals despite initiating all watch prompts due to non-wear. Candidate frailty-aligned metrics included steps (inactivity), cadence (slowness), longest walking bout (endurance), and activity intensity. Exploratory analyses suggested that comorbidity and polypharmacy aligned with lower activity and higher quality of life with greater intensity. GeRI's open-source architecture supports GA-aligned home monitoring and motivates refinements to improve data yield.

## Introduction

Older adults with frailty—a syndrome of reduced physiologic reserve—experience higher rates of cancer treatment toxicity, treatment interruption, hospitalization, and mortality (1). Comprehensive geriatric assessment (GA) identifies frailty and related vulnerabilities (multimorbidity, functional decline, polypharmacy) linked to poor outcomes (2), but remains underused due to logistical burden (3–6). Digital health technologies (DHTs) can capture functional features of frailty, including slowness, inactivity, and endurance. DHTs should match patient abilities, provide feedback, and minimize data collection burden (7). Proprietary hardware and closed pipelines, however, limit adaptability and reproducibility (8).

Open-source DHTs—publicly available code or hardware—can lower costs, improve interoperability, and enable community-driven innovation (9). Open methods for processing wearable data have also enabled new GA risk markers (10). We introduce the Geriatric Remote Initiative (GeRI), a modular, device-agnostic open-source platform for standardized collection and processing of frailty-aligned health metrics. We previously reported participatory design and simulated home use of an early GeRI prototype by older adults with cancer and caregivers (mean 25 days), achieving a mean System Usability Scale (SUS) score of 92.8/100 (11). After incorporating feedback and deploying GeRI on institution-managed on-premises servers, we report a proof-of-concept deployment in men with metastatic prostate cancer (mPC) to assess engagement, usability, data completeness, and clinical face validity of core metrics. Our primary contribution is the platform architecture and open methods, illustrated in a small mPC cohort.

Our long-term goal is to develop digital activity metrics aligned with frailty domains as remote indicators that support GA-informed risk stratification, treatment decisions, and digital endpoints for decentralized trials and chronic disease monitoring. In this proof-of-concept report, we (i) evaluate engagement and usability of GeRI in the home, (ii) describe technical/data capture performance, and (iii) report exploratory associations between device-derived activity metrics and GA domains. We define engagement as participant adherence to prompted platform tasks (responding to surveys, completing scale measurements, and initiating requested wearable monitoring). Engagement reflects participant behavior and is distinct from technical performance (prompt delivery and successful data capture) and analytic yield (data meeting prespecified validity criteria for analysis).

## Methods

### GeRI architecture and secure data flow

#### Hardware

**Figure 1:**
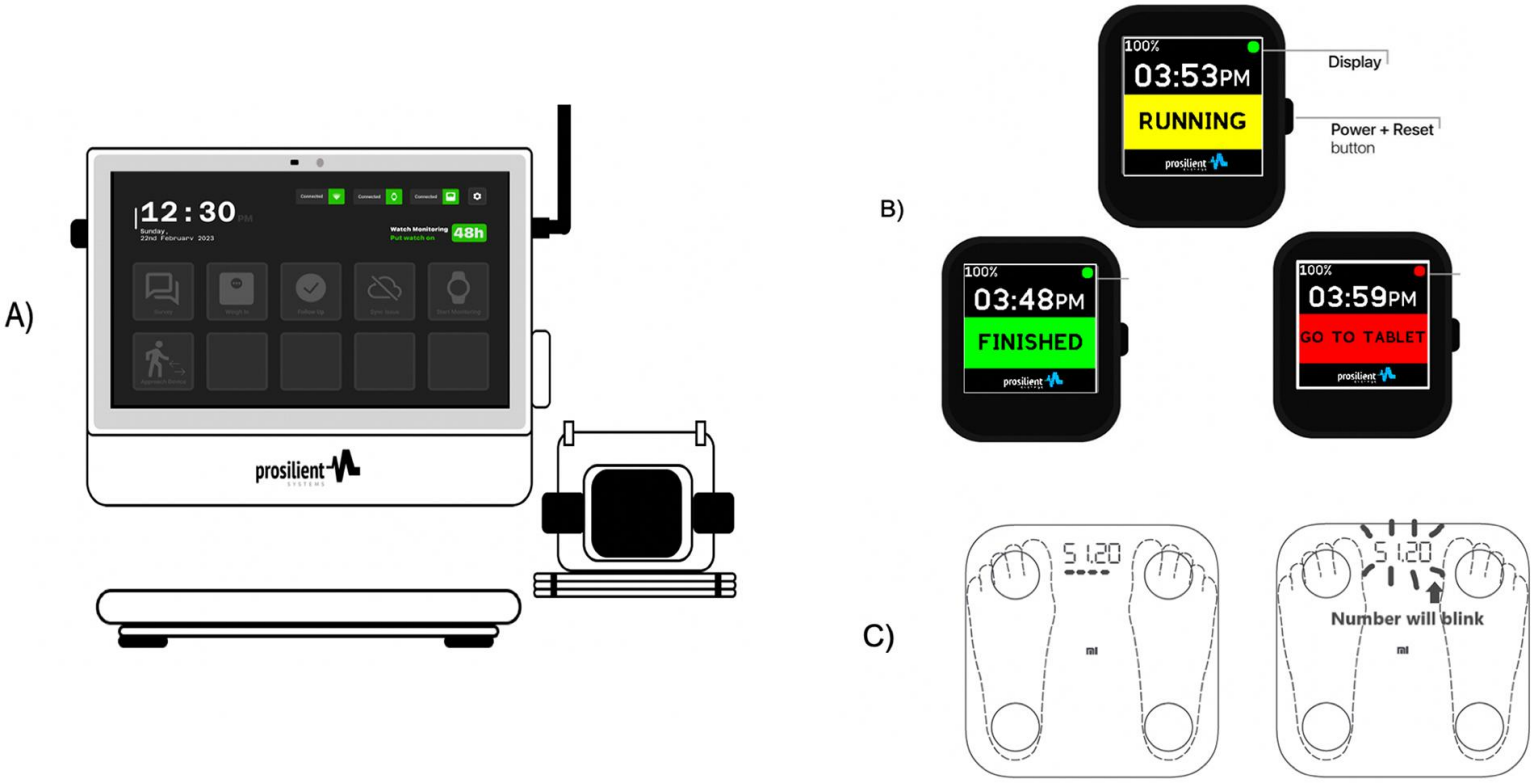
Home hardware and participant-facing displays for the GeRI platform. **(A)** Firozhub tabletop tablet (Raspberry Pi CM4) with Bangle.js 2 charging dock and paired Mi Body Composition Scale 2, as deployed in participants’ homes. **(B)** Bangle.js 2 watch screens shown during monitoring sessions, including the RUNNING, FINISHED, and GO TO TABLET prompts and the location of the power/reset button. **(C)** Mi Body Composition Scale 2 display showing correct foot placement and the blinking stable-weight indicator during measurement.

GeRI comprises an open-source wrist accelerometer (Bangle.js 2), a Bluetooth-enabled body composition scale (Mi Body Composition Scale 2), and a custom tabletop tablet (Firozhub) built on the Raspberry Pi Compute Module 4 (CM4) running a customized build of Raspberry Pi OS. Figure 1 illustrates the complete home setup, including the Firozhub tablet with the watch charging dock, the Bangle.js 2 wrist accelerometer, and the paired Bluetooth scale as deployed in participants’ homes.

#### On-device services

**Figure 2:**
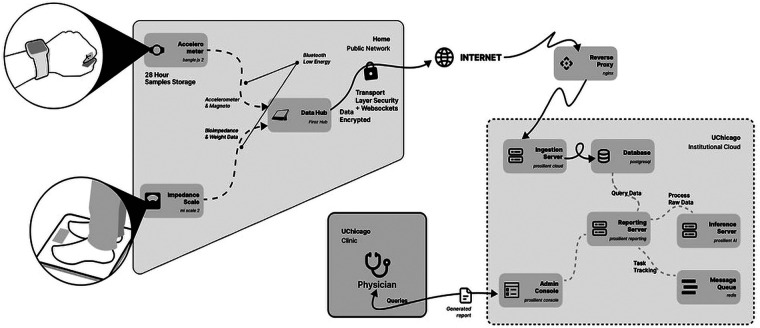
System architecture and data flow of the GeRI platform. At home, a wrist accelerometer (Bangle.js 2) and Bluetooth body composition scale (Mi Body Composition Scale 2) transmit data via Bluetooth Low Energy to the Raspberry Pi-based Firozhub tablet. The tablet encrypts payloads and transfers them over the home network using TLS with WebSockets. Within the University of Chicago on-premises cloud, an ingestion server receives data, a PostgreSQL database stores survey, and derived metrics; an inference server processes raw data; and a Redis message queue manages task tracking. A reporting server services clinician queries, and an admin console enables generation of de-identified reports. The deployment uses institution-managed infrastructure and open-source components without third-party vendor cloud involvement.

A system manager enforces time synchronization [network time protocol (NTP)] before other services launch. The Firoz Connectivity Manager pairs via Bluetooth with the wrist accelerometer and scale, timestamps incoming data, and passes events through the Firoz Cloud Connect service. Cloud Connect (i) creates a unique encrypted device identity on first boot, (ii) encodes messages using Protocol Buffers v3, (iii) buffers transmissions locally in an SQLite store until confirmed by the server, and (iv) retries with exponential back-off when offline. Device serial numbers are never transmitted; instead, a study identifier is derived from the unique device identity and provisioning date. All transport uses TLS 1.2 or higher encryption. The sequence of on-device services and message flow is summarized in Figure 2.

#### Cloud backend and analytics

Firoz Cloud Connect authenticates to an institution-hosted Prosilient Cloud instance (on-premises) over a secure WebSocket and ingests data into a PostgreSQL database, where an inference worker materializes step counts and Activity Index (AInd) from raw accelerometry. Audit logs, role-based access control, and encrypted backups are maintained according to institutional policy, and study teams can export de-identified datasets via a web portal. No commercial third-party clouds or vendor accounts are used at any stage. The data flow and backend components are illustrated in Figure 2, and the clinician portal in Supplementary Figure S1.

### Device-agnostic pipeline

Raw acceleration data (KX022-1020; ±8 g, 4,096 counts/g) were converted to g and summarized into 1-s epochs. Accelerometry was sampled at 10 Hz to balance signal fidelity with the Bangle.js 2's battery (∼50 h) and 8 MB storage constraints. Prior work has shown that reducing sampling from 50 to 10 Hz minimally impacts activity recognition accuracy (12). In addition, open-source 10 Hz step counters have successfully captured gait-related information while extending device runtime (13). Validation of the same Bangle.js 2 hardware at a low sampling rate demonstrated strong agreement with commercial step-counting devices in free-living conditions (14).

Device-agnostic accelerometry signal processing prioritized open methods to derive mobility metrics aligned with phenotypic frailty components linked previously to chemotherapy tolerance (15): *steps* (averaged over valid days; *inactivity*), *cadence* (“best pace” or the 90th-percentile steps/min; *slowness*), and longest walking bout or LWB (longest uninterrupted stepping sequence per valid day; *endurance*, capped at 2 h based on routine older adult LWBs) (16). We first applied a fixed bias correction derived from quiet-stationary intervals and set negative values within the noise floor to zero. Step events were then identified using the publicly available Salvi- and Casale-informed pipeline adapted for the wrist-worn 10 Hz Bangle.js 2 (17, 18). In brief, Casale's small classifier labels short windows as “moving” or “not moving” based on time domain features of the triaxial signal, and a Salvi-style peak detector is then applied within moving windows to identify regularly spaced, step-like peaks with physiologic timing constraints. Full implementation will be shared in a public GitHub repository.

We also explored AInd, an open-source variance-based measure of activity intensity originally developed for hip-worn accelerometers (19). We implemented AInd as described by Bai et al. on 1-s wrist epochs and treated it as an exploratory marker of overall activity intensity and sedentary variability in this context, inspired by our prior work in registry wrist datasets (10). AInd was computed as an hourly variance-based summary of wrist accelerometry during daytime wake hours (07:00–19:00), consistent with our prior hourly wrist accelerometry analyses in older adults showing that frailty-related differences are most apparent during daytime activity (20). For each 48-h window, the higher of the two 24-h summaries was retained.

*Non-wear* was flagged when axis SDs remained below thresholds on ≥2 axes over ≥90 min (21), with a 15-min padding before/after to avoid edge effects. For step-derived metrics, analyses included only valid 24-h intervals. Intervals were excluded if non-wear exceeded 1.5 h, defined using the published 90-min non-wear rule (22). Intervals with <300 steps were also removed because these values likely indicate that the device was not worn (23).

### Trigger and biometric sampling process

The platform was designed to prompt self-rated health (SRH) surveys (“excellent/very good/good/fair/poor”) as a low-burden global health measure (24) every 5–9 days on a rolling schedule. SRH prompts remained available for 48-h and could generate reminder/duplicate prompt instances if not completed. Fair/poor SRH responses triggered administration of the validated Patient-Reported Outcomes version of the Common Terminology Criteria for Adverse Events symptom surveys (graded 0–3 severity) (25). Watch wear session prompts (48 h) were conditional on SRH state (fair/poor) or random selection among good-or-better weeks. Therefore, prompt counts varied by participant and enabled sampling of habitual activity under differing symptom burden states rather than continuous background surveillance. Participants initiated data capture by pressing “Start monitoring” on the watch within a brief initiation window; uninitiated prompts expired after 48 h and were classified as non-adherence.

The 48-h monitoring window balanced participant burden, accelerometer memory constraints, and evidence that 2–3 monitoring days provide stable estimates of usual activity volume in older adults (26), allowing aggregation across the deployment period. Although prior studies used hip-worn devices, they primarily estimated the number of days required to average day-to-day activity variation, the same construct targeted by repeated 48-h wrist-based sessions. Participants were instructed to wear the watch only during monitoring periods and otherwise keep it on the charger. Each triggered window could yield up to two 24-h intervals depending on wear time and device performance.

Symptom grade ≥2 triggered repeat symptom surveys every 3 days until resolution (≤1). Weekly scale sessions recorded weight (kg) and impedance (*Ω*) under standardized conditions (morning, barefoot, before exercise). Weight and survey prompts were available for completion for up to 48 h before expiring and being logged as missing.

### Home deployment in a prostate cancer cohort

#### Eligibility and procedures

Men aged ≥65 years receiving androgen deprivation therapy were recruited from a single academic genitourinary oncology clinic. We restricted enrollment to this high-risk, clinically homogeneous population, which is central to our frailty research program, for this initial proof-of-concept deployment. The target sample size of 10 participants was chosen pragmatically to evaluate engagement, usability, and end-to-end data flow rather than to power formal hypothesis tests, consistent with recommendations showing that groups of about 10 users typically uncover ≥80% of usability in pilot testing (27). Inclusion required reliable home Wi-Fi and absence of cognitive impairment by a normal Mini-Cog score of 4 or 5 (28). Key exclusion criteria were early-stage cancer, hospice enrollment, severe dementia, or uncontrolled comorbidity. Participants completed a fixed ∼12-week home-monitoring phase. Written informed consent was obtained, and participants received a $100 gift card upon device return.

#### Geriatric assessment battery

GA was conducted at baseline and week 12 using the following instruments: Cumulative Illness Rating Scale-Geriatric (CIRS-G; comorbidity) (29), Cancer and Aging Research Group (CARG; toxicity risk) (30), Geriatric-8 (G8; nutrition; with a derived G8-SRH item) (31), Functional Assessment of Cancer Therapy—General (health-related quality of life) (32), and polypharmacy (active prescription count) (33). Prespecified change thresholds (week 12 vs. baseline) were G8 decline ≥1, CARG increase ≥1, CIRS-G increase ≥1, any G8-SRH downgrade, and FACT-G decline >3 (34).

### Outcomes and analysis

#### Primary outcomes

- *Engagement* was prespecified as ≥6/10 participants completing ≥50% of prompted tasks (surveys, scale sessions, and watch session initiations) within each 4-week block across the 12-week engagement period. The ≥50% threshold was defined during study design as a pragmatic feasibility criterion and has been used in genitourinary cancer electronic patient-reported outcomes (ePRO) studies (35). Engagement reflects participant adherence to prompted tasks and is distinct from technical performance. For each modality, we report end-to-end completeness (completed/scheduled prompts). For watch prompts, we additionally report aggregate participant-attributable initiation by excluding prompts with confirmed device-related failure during the initiation window. Prompts that could not be generated or displayed due to confirmed device malfunction were excluded from engagement denominators and summarized under technical/data capture outcomes.
- *Usability* was measured using the SUS, a validated 10-item questionnaire yielding a score from 0 to 100, with 68 commonly accepted as the cutoff for adequate usability (36).

#### Secondary/exploratory outcomes

- Technical/data-capture metrics:
  ○ For each data stream (SRH, symptoms, scale, and watch prompts), we quantified scheduled (platform-generated and displayed) versus completed prompts over the initial 12-week (84-day) engagement window. Scheduled counts represent platform-generated prompt instances that were successfully generated and displayed to participants, including reminder, duplicate, and w-linked re-prompting. A prompt was considered completed when a survey or smart-scale measurement was submitted or a watch-monitoring session was initiated (i.e., “Start monitoring” was pressed within the initiation window). For watch prompts, uninitiated monitoring sessions were attributed to protocol non-initiation unless a confirmed device-related failure occurred during the initiation window, in which case they were attributed to device-related failure. Accordingly, completed/scheduled reports reflect end-to-end completeness, whereas participant-attributable adherence excludes device-related failures from the denominator.
  ○ For accelerometry, we report (a) watch session initiation within 48 h during the 12-week engagement window and (b) recoverable and valid 24-h intervals over the full deployment-to-return interval. Initiation (“Start monitoring”) does not guarantee wear; initiated sessions could contribute 0 valid intervals.
- Device-derived metrics (steps, cadence, LWB, AInd) were examined in relation to baseline GA domains/symptoms, including impedance as an exploratory marker.

#### Statistical plan

Descriptive statistics [count, proportions, mean ± SD; median (IQR)] summarize demographics, usability, adherence, and overall data capture. For cross-sectional comparisons with GA domains, device-derived activity metrics were averaged across all valid 24-h intervals for each participant to reflect overall mobility during the deployment period. Baseline group differences were assessed using Wilcoxon/Kruskal–Wallis; change-vs.-no-change contrasts used two-sample *t*-tests. Weekly associations between activity metrics and patient-reported outcomes were modeled using generalized linear mixed models with a participant random intercept; fixed effects included SRH (0–5) and a composite pain + fatigue score (scaled 0–6). AInd trajectories were visualized by SRH/symptom slope (stable/improving vs. worsening). Mixed-effects models were fit at the interval level and used all available valid 24-h intervals for each metric; weeks without a valid interval contributed no accelerometry data and were not imputed. Analyses were performed in R v4.2.1. Given the small sample, p-values are nominal and exploratory; no multiplicity adjustments were applied (*α* = 0.05).

#### Ethics

The study was approved by the University of Chicago IRB (Protocol #22-0509). Procedures followed the Declaration of Helsinki, and all participants provided written informed consent.

## Results

### Participant demographics

Ten men with mPC were enrolled (median age = 73 years; range 66–79). Forty percent identified as a racial/ethnic minority, and 40% lived alone. Sixty percent met the CARG high-risk threshold (score > 5). Median duration of ongoing androgen suppression therapy at study entry was 3 months (range 2–4.5). Additional baseline details are shown in Table 1.

**Table 1:** Baseline demographics and clinical characteristics (*N* = 10).

Characteristic	*N* (%) or mean (SD)
Demographics	
Age (mean ± SD)	72.7 ± 4.8
Race	
White	6 (60.0%)
Black or African American	3 (30.0%)
Native American/Alaskan Native	1 (10.0%)
Ethnicity	
Not Hispanic or Latino	10 (100%)
Education	
Advanced degree	5 (50.0%)
Associate/bachelor's	3 (30.0%)
High school graduate	2 (20.0%)
Employment	
Full time	5 (50.0%)
Retired	5 (50.0%)
Living arrangement	
Spouse/partner	6 (60.0%)
Alone	4 (40.0%)
Prior and current cancer therapies	
Prior androgen suppression	4 (40.0%)
Current androgen receptor signaling inhibitor intensification	8 (80.0%)
Duration of androgen suppression at enrollment (months; median ± range)	3 (2–4.5)
History of prostatectomy	4 (40.0%)
History of prostate-directed radiation	4 (40.0%)
Current plan for radiation	5 (50.0%)
Baseline geriatric assessment measures	
Geriatric-8 < 14	2 (20.0%)
CIRS-G ≥ median of our sample	6 (60.0%)
FACT-G score	92.9 ± 12.0
CARG score >5	4 (40.0%)
Polypharmacy (>3 Rx/day)	7 (70.0%)
Health status as good or better	8 (80.0%)

Baseline demographics and clinical characteristics of *n* = 10 participants. Values are presented as *n* (%) unless otherwise specified. FACT-G, Functional Assessment of Cancer Therapy—General; CIRS-G, Cumulative Illness Rating Scale—Geriatric; CARG, Cancer and Aging Research Group.

### Engagement

Prespecified engagement was met: 9 of 10 participants achieved ≥50% completion of prompted interactions in each 4-week block across 12 weeks. One participant fell below 50% in a single 4-week block. Participant 1,821 initiated all watch-monitoring prompts but did not wear the device during prompted windows due to an unrelated hand injury. He was included in engagement and prompt-level initiation summaries but contributed 0 valid 24-h intervals and was excluded from accelerometry-derived outcomes due to non-wear.

### Usability

Across 10 participants, individual SUS total scores upon return of devices ranged from 57.5 to 100. One participant recorded the lowest score (57.5), while two participants scored 100. Overall, 8 of the 10 participants reported SUS scores of 75 or higher. Itemized SUS responses are summarized in Supplementary Figure S2.

### Data completeness and adherence

Over the first three contiguous 4-week blocks (84 days) from deployment, the platform generated 126 SRH prompts, 53 symptom survey blocks, 100 smart-scale prompts, and 62 watch-monitoring prompts (including reminder/duplicate and workflow-linked prompts). Participants completed 108/126 SRH prompts (86%), 46/53 symptom blocks (87%), and 83/100 scale prompts (83%), and initiated monitoring (“Start monitoring”) in 40/62 watch prompts (65%) (Table 2). For watch prompts, 22 were not initiated: 14 due to participant non-initiation, and 8 due to device malfunction during the prompt window (Table 2). Excluding the eight prompts with confirmed device malfunction during the prompt window, participant-attributable watch initiation was 40/54 (74%). For surveys and scale prompts, incomplete prompts reflected participant non-completion (prompts were displayed and allowed to expire without submission).

**Table 2:** Participant-level GeRI prompt completion (end-to-end completeness) during the 12-week engagement window.

Participant	SRH scheduled/completed	SRH incomplete (participant/device)	Symptom blocks scheduled/completed	Symptom incomplete (participant/device)	Scale scheduled/completed	Scale incomplete (participant/device)	Watch scheduled/initiated	Watch incomplete (participant/device)
2ba4	16/16	0/0	5/5	0/0	11/11	0/0	6/4	2/0
1,821**^a^**	11/9	2/0	10/9	1/0	11/9	2/0	5/5	0/0
50a8	16/16	0/0	1/1	0/0	11/11	0/0	6/5	1/0
996d	13/10	3/0	1/1	0/0	10/8	2/0	4/3	0/1
CFD1	11/9	2/0	5/3	2/0	9/8	1/0	7/5	2/0
5cd9	14/12	2/0	6/6	0/0	11/9	2/0	6/2	0/4
E5b2	17/15	2/0	7/4	3/0	11/8	3/0	7/2	2/3
03a7	12/7	5/0	6/6	0/0	11/6	5/0	7/3	4/0
2,863	9/9	0/0	8/7	1/0	9/8	1/0	9/8	1/0
7d78	7/5	2/0	4/4	0/0	6/5	1/0	5/3	2/0
Total	126/108	18/0	53/46	7/0	100/83	17/0	62/40	14/8

“Scheduled” counts reflect prompt instances that were successfully generated and displayed during the 12-week engagement window, including reminder or workflow-linked prompts. “Completed” indicates a submitted survey response, transmitted smart-scale measurement, or logged watch session initiation (“Start monitoring”). “Incomplete” indicates a delivered prompt without completion and is classified as participant-related when a shown prompt expired without completion and device-related when a confirmed malfunction during the prompt window prevented completion. Table 2 presents end-to-end completeness by participant; participant-attributable adherence is summarized in aggregate in the Results section.

^a^ Participant (1,821) initiated monitoring but did not wear the device during prompted windows; therefore, no valid 24-h accelerometry intervals were obtained.

### Device-derived activity, geriatric risk, and symptom burden

Devices remained in the field beyond 12 weeks to accommodate logistics and symptom-triggered sampling; therefore, accelerometer completeness was summarized over the full deployment-to-return interval (median 109 days; IQR 96–141). Across this period, nine analyzable participants received 80 watch prompts and initiated 40 monitoring sessions; one additional session had indeterminate initiation status and was excluded from denominator calculations. Each confirmed initiation corresponded to a 48-h monitoring window, potentially contributing up to 80 potentially recoverable 24-h intervals (40 × 2) for step-derived analyses. This maximum does not account for Participant 1,821, who initiated all prompts but did not wear the device. After step interval validity filtering, analytic yield was limited: 43 valid 24-h intervals were available for step-derived metrics (median 4 per participant, IQR 2–7), implying 43/80 (54%) usable intervals and 37/80 (46%) unusable intervals relative to the maximum recoverable from confirmed initiations.

Aggregating data across valid 24-h intervals from nine analyzable devices, the mean daily activity was 8,685 ± 5,754 steps, cadence 40.5 ± 18.2 steps/min, LWB 1,541 ± 979 steps, and AInd 96,239 ± 39,098. In exploratory tabulations of 24-h accelerometer AInd intervals across the full deployment-to-return interval by overall SRH slope (Supplementary Table S1), participants with decreasing SRH (poorer health) also contributed fewer total intervals than those with increasing or stable SRH. Supplementary Figure S3 visualizes cross-sectional differences in steps, cadence, LWB, and AInd across GA strata; directions mirror Supplementary Table S2. Cross-sectionally, polypharmacy and higher multimorbidity (CIRS-G > median) were associated with fewer steps, shorter LWBs, and lower AInd; poorer nutrition (G8 < 14) and higher toxicity risk (CARG >5) showed similar directions. In contrast, better quality of life (FACT-G > median) aligned with more steps and higher AInd. SRH comparisons were mixed (slightly higher steps but lower cadence and LWB for “good/excellent”); cadence effects were inconsistent overall. Longitudinal “worse vs. not” contrasts indicated decreases in steps, cadence, or LWB when G8, FACT-G, or SRH declined, whereas CARG change did not show a consistent decrease pattern. Impedance showed no consistent associations with GA domains. Trajectory analyses of AInd vs. SRH (0–5) and summed symptom pain and fatigue (0–6 total) slopes were not significant (Supplementary Figure S4). All inferential results are exploratory with nominal *p*-values and no multiplicity.

## Discussion

In this proof-of-concept deployment, GeRI met prespecified engagement and usability targets for home monitoring in older men with mPC. Although accelerometer sufficiency was not a primary endpoint, only 43 of 80 potentially recoverable 24-h intervals met validity criteria, indicating substantial attrition between session initiation and analyzable accelerometry due to non-wear during initiated sessions and device-level failures. As such, accelerometry data capture performance was limited in this prototype configuration.

Rather than replicating a full GA, GeRI provides GA-aligned monitoring that can inform GA-based care. It focuses on a small set of pragmatic measures—SRH survey, symptom triggers, device-agnostic accelerometry, and weight/impedance—that map to core GA domains including functional status, symptom burden, and nutritional risk. This approach builds on prior mobile health efforts such as the NIH-funded HOPE pilot (37), which tested symptom-triggered alerts using the open-source Beiwe platform paired with proprietary Fitbit devices in 10 patients with advanced gynecologic cancers. Unlike HOPE's reliance on closed commercial wearables and continuous smartphone connectivity, GeRI processes GA-aligned signals through open, reproducible pipelines running on institution-managed infrastructure and low-cost, open-source-compatible hardware. This design targets barriers related to device cost, network variability, and digital access—issues particularly relevant for older adults and safety net populations—and aims to make GA-informed digital monitoring more deployable in resource-limited geriatric oncology settings.

GeRI's trigger logic (SRH → symptom → accelerometry) and open accelerometry processing align with recommendations from the FDA-endorsed Clinical Trials Transformation Initiative (CTTI) (38). By employing prespecified, standardized, remotely captured endpoints with clear operational definitions (e.g., valid-day rules, feature windows, sampling plans), GeRI provides inspection-ready data provenance through audit logs and version-controlled pipelines, supporting reproducibility and regulatory oversight. Complementing this, the FDA's DHT guidance (38–40) outlines expectations for fit-for-purpose selection, verification and validation, prespecification of endpoints, algorithm version control, and data integrity in remote acquisition—principles that GeRI's reproducible pipelines are designed to follow, although addressing sample recording issues, missingness, and full endpoint-level validation remains a goal for future work. Finally, embedding validated patient-reported toxicity measures strengthens measurement validity in oncology decentralized trials (41).

Integrating standard geriatric assessments with sensor-derived activity metrics captures the spectrum of activity and frailty in men with mPC. The selected features map to recognized frailty constructs relevant to treatment tolerance—steps (inactivity), cadence (slowness), and longest walking bout (endurance) (15). Our data concur: Higher comorbidity scores and polypharmacy were associated with fewer steps, shorter LWBs, and slower cadence; lower G8 scores tracked similar declines, underscoring the functional toll of multimorbidity. Beyond step counts, the open-source AInd, which is sensitive to light-intensity movement and sedentary variability in older adults (19), correlated with better quality of life and lower comorbidity and varied alongside symptom burden in some participants. While hypothesis-generating, these patterns are consistent with geriatric vulnerability and suggest promising candidate digital measures for future exploration of resilience monitoring.

We use repeated 48-h monitoring windows to balance reliability with burden. Reliability studies suggest that ∼2–4 days often suffice to estimate total activity in older adults (42). Shorter windows can under-sample daily variability but could achieve acceptable group-level reliability when repeated (43); individual-level inference requires dedicated validation. Furthermore, scale impedance-derived measures, largely reflective of hydration and adiposity, did not associate with GA parameters—likely because commercial devices lack the precision to detect early sarcopenia (44)—although other wearable methods calibrated to absorptiometry may address this gap (45).

This study has several limitations. The sample was intentionally small and disease-specific, focusing on a high-risk group central to the mPC clinic. Ten men were enrolled; nine contributed usable accelerometry data. These data were processed into step-derived metrics, yielding 43 valid 24-h intervals, which still required validation on the Bangle.js 2 hardware. Accelerometry was captured only during symptom-triggered 48-h windows rather than via continuous wear over 12 weeks, a pragmatic compromise driven by participant burden, the 10 Hz sampling rate, and device memory and battery constraints. Missingness reflected both protocol non-initiation and device-related issues. During the 12-week engagement window, 22 of 62 watch-monitoring prompts were not initiated; 14 were attributable to participant/protocol non-initiation (prompts expired without initiation) and 8 to confirmed device-related failure during the prompt window. Among initiated sessions, additional losses from intermittent data capture and non-wear reduced the yield of analyzable intervals. Backend log review suggested that expected accelerometer volume was reduced by ∼46%–48% relative to the 80 potentially recoverable 24-h intervals over the full deployment-to-return interval. Device replacement or software reburn improved these issues later in deployment but constrained early data capture. The platform also occasionally generated duplicate SRH prompt instances (e.g., reminder/workflow-linked prompts), such that scheduled prompt counts could exceed the intended cadence in some periods. Generalizability to other cancers, to women, and to more diverse care settings is limited, and the digital activity metrics cannot yet be definitively evaluated as clinical endpoints.

Although overall engagement with GeRI was high, accelerometer completeness was further limited by several technical factors. The Bangle.js 2's 8-MB memory, combined with 10 Hz sampling, required frequent off-loading, and intermittent memory card buffering via the Firozhub tablet disrupted cloud transmission. Non-adherence also contributed, including prompt expirations and one participant initiating multiple sessions without wearing the watch. Valid intervals were sparser among participants whose SRH declined, suggesting that symptom-related non-initiation also played a role. These feasibility-impacting issues indicate that the current GeRI configuration is not yet sufficient for endpoint-level activity monitoring and will require improved buffering, direct-to-cloud transmission, and real-time non-wear prompting. Privacy-driven, key-based authentication also blocked bidirectional data flow, preventing patients from viewing real-time metrics, which is a known barrier to sustained digital health use (7). Deploying GeRI with an established open-source or commercial-grade accelerometer with larger memory on institutionally approved commercial clouds or enabling on device trend visualization could restore feedback while preserving security. Despite these constraints, GeRI's open-source framework remains a scalable foundation; refinements in usability, data retention, and bidirectional communication will further strengthen its value in geriatric oncology.

Future work will finalize an operations package aligned with CTTI recommendations (38, 39) and incorporate the Digital Medicine Society's V3 framework—sensor verification, analytical validation, and clinical validation of device-derived activity metrics (46). Multicenter studies using more robust commercial sensors (e.g., Samsung Galaxy Watch) and continuous wear schemes will allow explicit prespecification of sampling requirements, valid-day thresholds, and missing data strategies needed for digital activity endpoints. With these refinements, GeRI's open, auditable architecture can support scalable, equitable deployment of geriatric-aligned digital endpoints in oncology and aging research. Our ongoing Geriatric Assessment and Technology Evaluation in Prostate Cancer (ProsGATE) study (NCT06215508) will test integration with a commercially validated Samsung Galaxy Watch that has demonstrated success with geriatric-specific analytics (47). ProsGATE will incorporate prospective activity and body composition monitoring, real-time adherence monitoring, and adherence-based monetary rewards to provide a practicable route to large-scale deployment and wearable data quality (47).

## Conclusion

GeRI is a user-friendly, open-source platform for remote geriatric-aligned monitoring in older adults with metastatic prostate cancer. This proof-of-concept deployment met engagement and usability metrics but also showed that the current open-source accelerometer configuration and intermittent, trigger-based sampling may limit data density for longitudinal monitoring. Next steps will focus on improving data capture feasibility by combining multicenter evaluation with prespecified sampling requirements, continuous or higher-frequency wear schemes, and technical enhancements such as direct-to-cloud transmission and real-time wear detection. Integration of commercial wearables will address current accelerometry limitations while preserving GeRI's open, auditable pipelines. The ongoing ProsGATE trial (NCT06215508) will test this strategy, enabling scalable deployment and supporting future oncology trials.

## Author's note

Presented as Meeting Abstract at the 2024 ASCO Annual Meeting in Chicago and as Posters at the 14th and 15th International Conference on Frailty and Sarcopenia Research in New Mexico, USA, and Toulouse, France, respectively.

## Code availability

The GeRI platform is developed as an open-source system, with source code distributed across public GitHub repositories. The public meta-repository for GeRI (https://github.com/ProsilientSystems/prosilient-geri) provides architecture documentation, data-flow diagrams, build instructions, and links to all platform components. At the time of publication, core cloud-side services and analytics components of the GeRI platform are publicly available. Additional tablet-side services and Bangle.js2 firmware will be released in public repositories no later than the time of publication.

## Data Availability

The datasets generated during this study contain human participant data and are not publicly available. De-identified data may be made available to qualified investigators upon reasonable request to the corresponding author, subject to institutional review board approval and execution of a data use agreement.

## References

[1] Flannery MA Culakova E Canin BE Peppone L Ramsdale E Mohile SG. Understanding treatment tolerability in older adults with cancer. J Clin Oncol. (2021) 39(19):2150–63. 10.1200/JCO.21.00195 34043433 PMC8238902

[2] Dale W Klepin HD Williams GR Alibhai SMH Bergerot C Brintzenhofeszoc K Practical assessment and management of vulnerabilities in older patients receiving systemic cancer therapy: ASCO guideline update. J Clin Oncol. (2023) 41(26):4293–312. 10.1200/JCO.23.00933 37459573 PMC12803700

[3] Dale W Williams GR MacKenzie AR Soto-Perez-de-Celis E Maggiore RJ Merrill JK How is geriatric assessment used in clinical practice for older adults with cancer? A survey of cancer providers by the American Society of Clinical Oncology. JCO Oncol Pract. (2021) 17(6):336–44. 10.1200/OP.20.00442 33064058 PMC8462667

[4] Pringle S Ko EM Doherty M Smith AJB. Addressing transportation barriers in oncology: existing programs and new solutions. Support Care Cancer. (2024) 32(5):317. 10.1007/s00520-024-08514-2 38684580 PMC11058971

[5] Neo J Fettes L Gao W Higginson IJ Maddocks M. Disability in activities of daily living among adults with cancer: a systematic review and meta-analysis. Cancer Treat Rev. (2017) 61:94–106. 10.1016/j.ctrv.2017.10.006 29125982

[6] Bevans M Sternberg EM. Caregiving burden, stress, and health effects among family caregivers of adult cancer patients. JAMA. (2012) 307(4):398–403. 10.1001/jama.2012.29 22274687 PMC3304539

[7] Hasnan S Aggarwal S Mohammadi L Koczwara B. Barriers and enablers of uptake and adherence to digital health interventions in older patients with cancer: a systematic review. J Geriatr Oncol. (2022) 13(8):1084–91. 10.1016/j.jgo.2022.06.004 35752605

[8] Montgomery SM Nair N Chen P Dikker S. Introducing EmotiBit, an open-source multi-modal sensor for measuring research-grade physiological signals. Sci Talks. (2023) 6:100181. 10.1016/j.sctalk.2023.100181

[9] Paton C Braa J Muhire A Marco-Ruiz L Kobayashi S Fraser H Open source digital health software for resilient, accessible and equitable healthcare systems. Yearb Med Inform. (2022) 31(1):67–73. 10.1055/s-0042-1742508 35654431 PMC9719763

[10] Shi C Babiker N Urbanek JK Grossman RL Huisingh-Scheetz M Rzhetsky A. Free-living wrist and hip accelerometry forecast cognitive decline among older adults without dementia over 1- or 5-years in two distinct observational cohorts. NPJ Aging. (2022) 8(1):7. 10.1038/s41514-022-00087-w 35927250 PMC9170733

[11] Mir N Curry G Lee NK Szmulewitz RZ Huisingh-Scheetz M. A usability and participatory design study for GeRI, an open-source, remote cancer treatment toxicity and frailty monitoring platform for older adults. J Geriatr Oncol. (2023) 15:101595. 10.1016/j.jgo.2023.101595 37487857 PMC10800671

[12] Yamane T Kimura M Morita M. Effects of sampling frequency on human activity recognition with machine learning aiming at clinical applications. Sensors (Basel). (2025) 25(12):3780. 10.3390/s25123780 40573667 PMC12196717

[13] Straczkiewicz M Huang EJ Onnela J-P. A “one-size-fits-most” walking recognition method for smartphones, smartwatches, and wearable accelerometers. NPJ Digit Med. (2023) 6(1):29. 10.1038/s41746-022-00745-z 36823348 PMC9950089

[14] Ravanelli N Lefebvre K Brough A Paquette S Lin W. Validation of an open-source smartwatch for continuous monitoring of physical activity and heart rate in adults. Sensors (Basel). (2025) 25(9):2926. 10.3390/s25092926 40363363 PMC12074211

[15] Cay G Sada YH Dehghan Rouzi M Uddin Atique MM Rodriguez N Azarian M Harnessing physical activity monitoring and digital biomarkers of frailty from pendant based wearables to predict chemotherapy resilience in veterans with cancer. Sci Rep. (2024) 14(1):2612. 10.1038/s41598-024-53025-z 38297103 PMC10831115

[16] Ustad A Sverdrup K Tangen GG Døhl Ø Vereijken B Thingstad P Daily physical activity in older adults across levels of care: the HUNT Trondheim 70+study. Eur Rev Aging Phys Act. (2024) 21(1):20. 10.1186/s11556-024-00355-6 39014310 PMC11253329

[17] Salvi D Velardo C Brynes J Tarassenko L. An optimised algorithm for accurate steps counting from smart-phone accelerometry. Annu Int Conf IEEE Eng Med Biol Soc. (2018) 2018:4423–7. 10.1109/EMBC.2018.8513319 30441333

[18] Casale P Pujol O Radeva P. Human Activity Recognition from Accelerometer Data Using a Wearable Device. Pattern Recognition and Image Analysis. Berlin: Springer (2011).

[19] Bai J Di C Xiao L Evenson KR LaCroix AZ Crainiceanu CM An activity index for raw accelerometry data and its comparison with other activity metrics. PLoS One. (2016) 11(8):e0160644. 10.1371/journal.pone.0160644 27513333 PMC4981309

[20] Huisingh-Scheetz M Wroblewski K Kocherginsky M Huang E Dale W Waite L The relationship between physical activity and frailty among U.S. older adults based on hourly accelerometry data. J Gerontol A Biol Sci Med Sci. (2018) 73(5):622–9. 10.1093/gerona/glx208 29106478 PMC5905616

[21] van Hees VT Renström F Wright A Gradmark A Catt M Chen KY Estimation of daily energy expenditure in pregnant and non-pregnant women using a wrist-worn tri-axial accelerometer. PLoS One. (2011) 6(7):e22922. 10.1371/journal.pone.0022922 21829556 PMC3146494

[22] Choi L Ward SC Schnelle JF Buchowski MS. Assessment of wear/nonwear time classification algorithms for triaxial accelerometer. Med Sci Sports Exerc. (2012) 44(10):2009–16. 10.1249/MSS.0b013e318258cb36 22525772 PMC3443532

[23] Tudor-Locke C Schuna JM Jr. Barreira TV Mire EF Broyles ST Katzmarzyk PT Normative steps/day values for older adults: NHANES 2005–2006. J Gerontol A Biol Sci Med Sci. (2013) 68(11):1426–32. 10.1093/gerona/glt116 23913932

[24] Giri S Mir N Al-Obaidi M Clark D Kenzik KM McDonald A Use of single-item self-rated health measure to identify frailty and geriatric assessment-identified impairments among older adults with cancer. Oncologist. (2022) 27(1):e45–52. 10.1093/oncolo/oyab020 35305105 PMC8842332

[25] Basch E Becker C Rogak LJ Schrag D Reeve BB Spears P Composite grading algorithm for the national cancer institute’s patient-reported outcomes version of the common terminology criteria for adverse events (PRO-CTCAE). Clin Trials. (2021) 18(1):104–14. 10.1177/1740774520975120 33258687 PMC7878323

[26] Kocherginsky M Huisingh-Scheetz M Dale W Lauderdale DS Waite L. Measuring physical activity with hip accelerometry among U.S. older adults: how many days are enough? PLoS One. (2017) 12(1):e0170082. 10.1371/journal.pone.0170082 28081249 PMC5231361

[27] Faulkner L. Beyond the five-user assumption: benefits of increased sample sizes in usability testing. Behav Res Methods Instrum Comput. (2003) 35(3):379–83. 10.3758/BF03195514 14587545

[28] Abayomi SN Sritharan P Yan E Saripella A Alhamdah Y Englesakis M The diagnostic accuracy of the Mini-Cog screening tool for the detection of cognitive impairment—a systematic review and meta-analysis. PLoS One. (2024) 19(3):e0298686. 10.1371/journal.pone.0298686 38483857 PMC10939258

[29] Miller MD Paradis CF Houck PR Mazumdar S Stack JA Rifai AH Rating chronic medical illness burden in geropsychiatric practice and research: application of the cumulative illness rating scale. Psychiatry Res. (1992) 41(3):237–48. 10.1016/0165-1781(92)90005-N 1594710

[30] Hurria A Togawa K Mohile SG Owusu C Klepin HD Gross CP Predicting chemotherapy toxicity in older adults with cancer: a prospective multicenter study. J Clin Oncol. (2011) 29(25):3457–65. 10.1200/JCO.2011.34.7625 21810685 PMC3624700

[31] Bellera CA Rainfray M Mathoulin-Pélissier S Mertens C Delva F Fonck M Screening older cancer patients: first evaluation of the G-8 geriatric screening tool. Ann Oncol. (2012) 23(8):2166–72. 10.1093/annonc/mdr587 22250183

[32] Overcash J Extermann M Parr J Perry J Balducci L. Validity and reliability of the FACT-G scale for use in the older person with cancer. Am J Clin Oncol. (2001) 24(6):591–6. 10.1097/00000421-200112000-00013 11801761

[33] Ramsdale E Mohamed M Yu V Otto E Juba K Awad H Polypharmacy, potentially inappropriate medications, and drug-drug interactions in vulnerable older adults with advanced cancer initiating cancer treatment. Oncologist. (2022) 27(7):e580–8. 10.1093/oncolo/oyac053 35348764 PMC9255971

[34] Brucker PS Yost K Cashy J Webster K Cella D. General population and cancer patient norms for the functional assessment of cancer therapy-general (FACT-G). Eval Health Prof. (2005) 28(2):192–211. 10.1177/0163278705275341 15851773

[35] Yurdakul O Alan A Krauter J Korn S Gust K Shariat SF Impact of immigration background on feasibility of electronic patient-reported outcomes in advanced urothelial cancer patients. Health Qual Life Outcomes. (2024) 22(1):107. 10.1186/s12955-024-02325-z 39696509 PMC11657869

[36] Lewis JR. The system usability scale: past, present, and future. Int J Hum Comput Interact. (2018) 34(7):577–90. 10.1080/10447318.2018.1455307

[37] Wright AA Raman N Staples P Schonholz S Cronin A Carlson K The HOPE pilot study: harnessing patient-reported outcomes and biometric data to enhance cancer care. JCO Clin Cancer Inform. (2018) 2:1–12. 10.1200/CCI.17.00149 30652585 PMC6556148

[38] Clinical Trials Transformation Initiative (CTTI). Decentralized Clinical Trials Recommendations. (2021). Available online at: https://ctti-clinicaltrials.org/wp-content/uploads/2021/06/CTTI_DCT_Recs.pdf (accessed September 7, 2025). 10.4415/ANN_11_01_04 21430332

[39] Clinical Trials Transformation Initiative (CTTI). Supporting Decentralized Approaches to Clinical Trials: Recommendations. (2021). Available online at: https://ctti-clinicaltrials.org/wp-content/uploads/2021/07/CTTI_Supporting_Decentralized_Approaches_Recommendations.pdf (accessed September 7, 2025). 10.4415/ANN_11_01_04 21430332

[40] U.S. Food and Drug Administration. Digital Health Technologies for Remote Data Acquisition in Clinical Investigations: Guidance for Industry, Investigators, and Other Stakeholders. Silver Spring, MD: FDA (2023). Available online at: https://www.fda.gov/regulatory-information/search-fda-guidance-documents/digital-ealth-technologies-remote-data-acquisition-clinical-investigations (accessed September 7, 2025).

[41] Dueck AC Mendoza TR Mitchell SA Reeve BB Castro KM Rogak LJ Validity and reliability of the US national cancer institute’s patient-reported outcomes version of the common terminology criteria for adverse events (PRO-CTCAE). JAMA Oncol. (2015) 1(8):1051–9. 10.1001/jamaoncol.2015.2639 26270597 PMC4857599

[42] Hart TL Swartz AM Cashin SE Strath SJ. How many days of monitoring predict physical activity and sedentary behaviour in older adults? Int J Behav Nutr Phys Act. (2011) 8(1):62. 10.1186/1479-5868-8-62 21679426 PMC3130631

[43] Aadland E Ylvisåker E. Reliability of objectively measured sedentary time and physical activity in adults. PLoS One. (2015) 10(7):e0133296. 10.1371/journal.pone.0133296 26192184 PMC4508000

[44] Frija-Masson J Mullaert J Vidal-Petiot E Pons-Kerjean N Flamant M d'Ortho MP. Accuracy of smart scales on weight and body composition: observational study. JMIR Mhealth Uhealth. (2021) 9(4):e22487. 10.2196/22487 33929337 PMC8122302

[45] Bennett JP Liu YE Kelly NN Quon BK Wong MC McCarthy C Next-generation smart watches to estimate whole-body composition using bioimpedance analysis: accuracy and precision in a diverse, multiethnic sample. Am J Clin Nutr. (2022) 116(5):1418–29. 10.1093/ajcn/nqac200 35883219 PMC11530365

[46] Goldsack JC Coravos A Bakker JP Bent B Dowling AV Fitzer-Attas C Verification, analytical validation, and clinical validation (V3): the foundation of determining fit-for-purpose for biometric monitoring technologies (BioMeTs). NPJ Digit Med. (2020) 3(1):55. 10.1038/s41746-020-0260-4 32337371 PMC7156507

[47] Smail EJ Alpert JM Mardini MT Kaufmann CN Bai C Gill TM Feasibility of a smartwatch platform to assess ecological mobility: real-time online assessment and mobility monitor. J Gerontol A Biol Sci Med Sci. (2023) 78(5):821–30. 10.1093/gerona/glad046 36744611 PMC10172974

