# Supplementary material for: An in-home engagement and usability study of GeRI: an open-source platform for remote symptom assessment and wearable activity monitoring in men with prostate cancer
Source: Front Digit Health. 2026 Mar 20;8:1700852. doi: 10.3389/fdgth.2026.1700852 (PMC13047109; doi:10.3389/fdgth.2026.1700852)
Supplement: Supplementary file 1 [file Datasheet1.docx]

# Supplementary Materials

## Figure S1: GeRI clinician portal (Prosilient Console Client; on-premises deployment)

*Description: Screenshot of the clinician dashboard used to monitor GeRI participation and data capture. The left panel filters data sources (wearable, scale, survey) and outputs, while the main table summarizes device metadata and connection status. Example entries are pseudonymous.*

## Figure S2. Distribution of System Usability Scale item responses for the GeRI platform (n = 10).

##
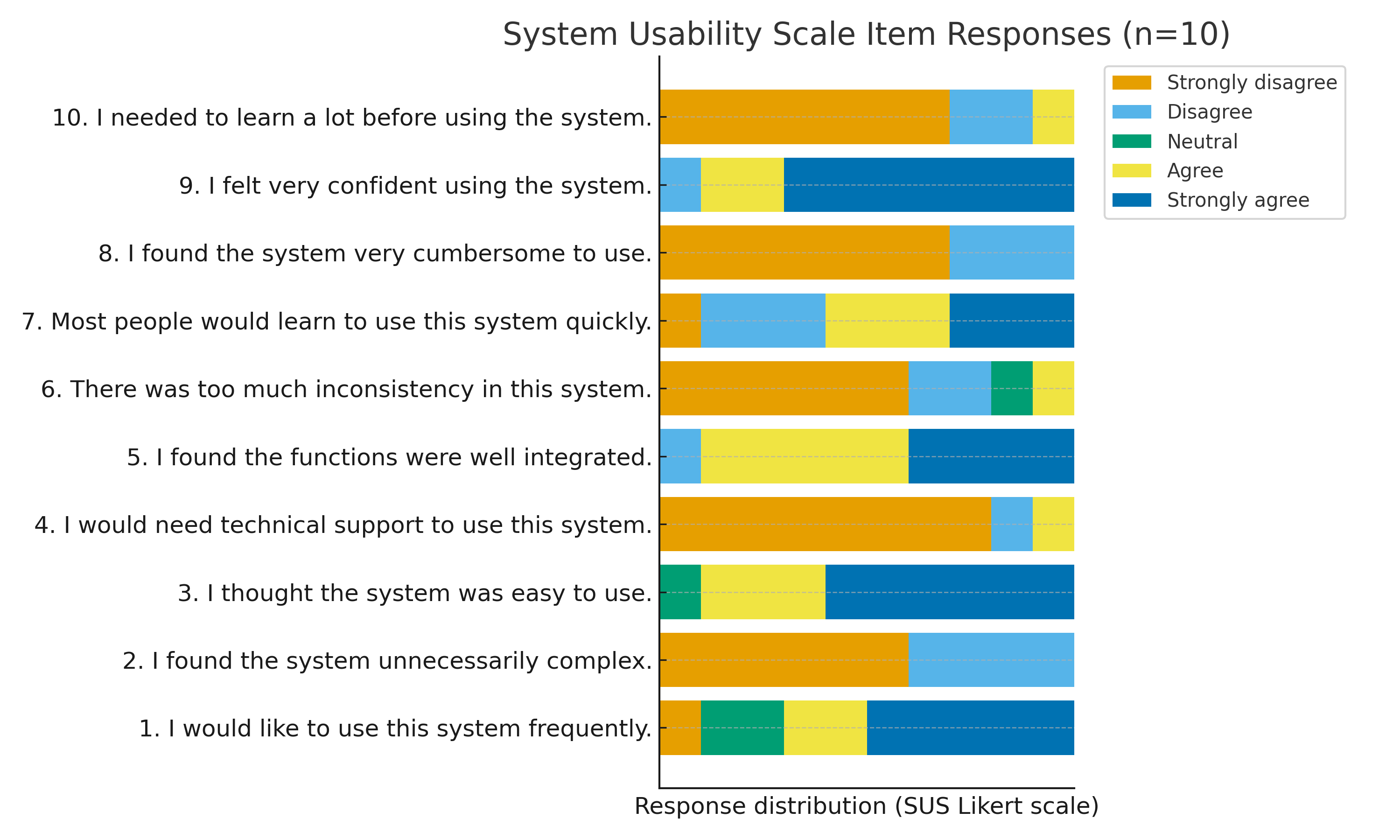


## *Description: Horizontal stacked bar chart showing the distribution of Likert responses (strongly disagree, disagree, neutral, agree, strongly agree) for each of the 10 System Usability Scale (SUS) items completed by participants using the GeRI platform. Each bar represents the percentage of respondents selecting each response category for that item; item wording is shown on the y-axis. Higher response categories indicate better perceived usability for positively worded items (1, 3, 5, 7, 9) and worse perceived usability for negatively worded items (2, 4, 6, 8, 10).*

**Figure S3 (A-D). Associations Between Baseline Geriatric Assessment Domains and Accelerometry-Derived Metrics**

A)


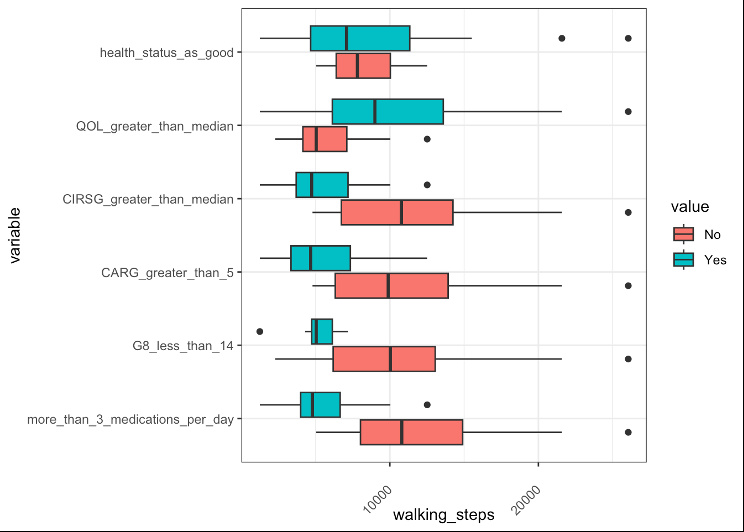


B)


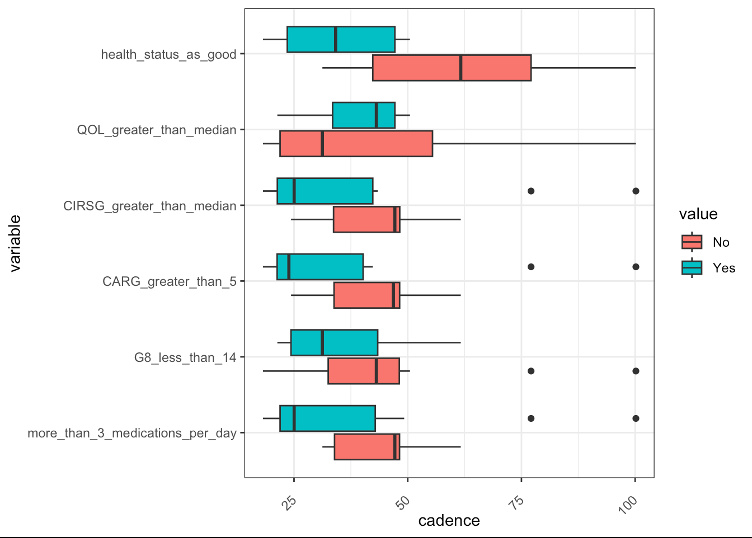


## C)
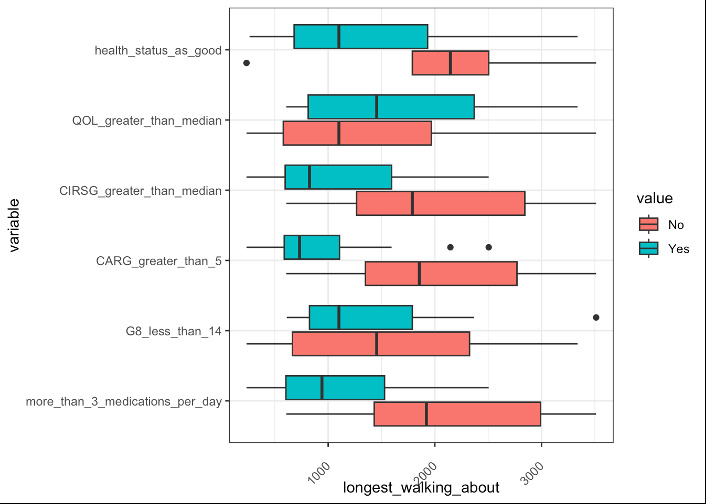


D)


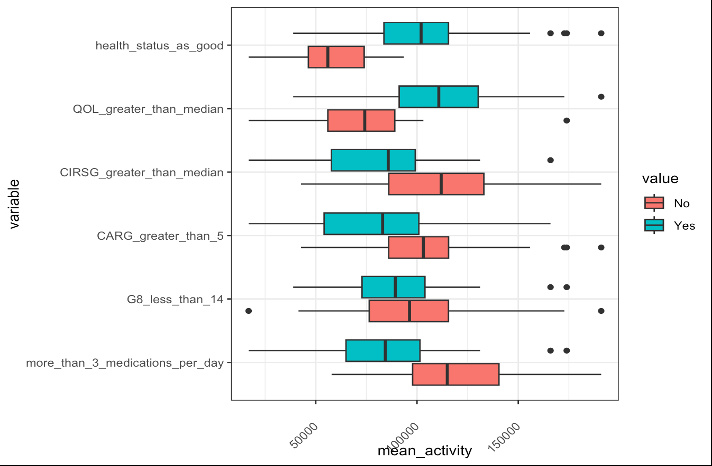


*Description: Device-derived mobility metrics across geriatric strata. Group differences for (A) steps/day, (B) cadence (90th-percentile steps/min), (C) longest walking bout (LWB), and (D) Activity Index (AInd) by CIRS-G, G8, CARG, polypharmacy, and FACT-G strata. Points show means; bars show 95% CIs; analyses exploratory.*

## Figure S4 (A & B): Composite Symptom Score Change, Self-Rated Health, and Activity Index (AInd) % Change

A)


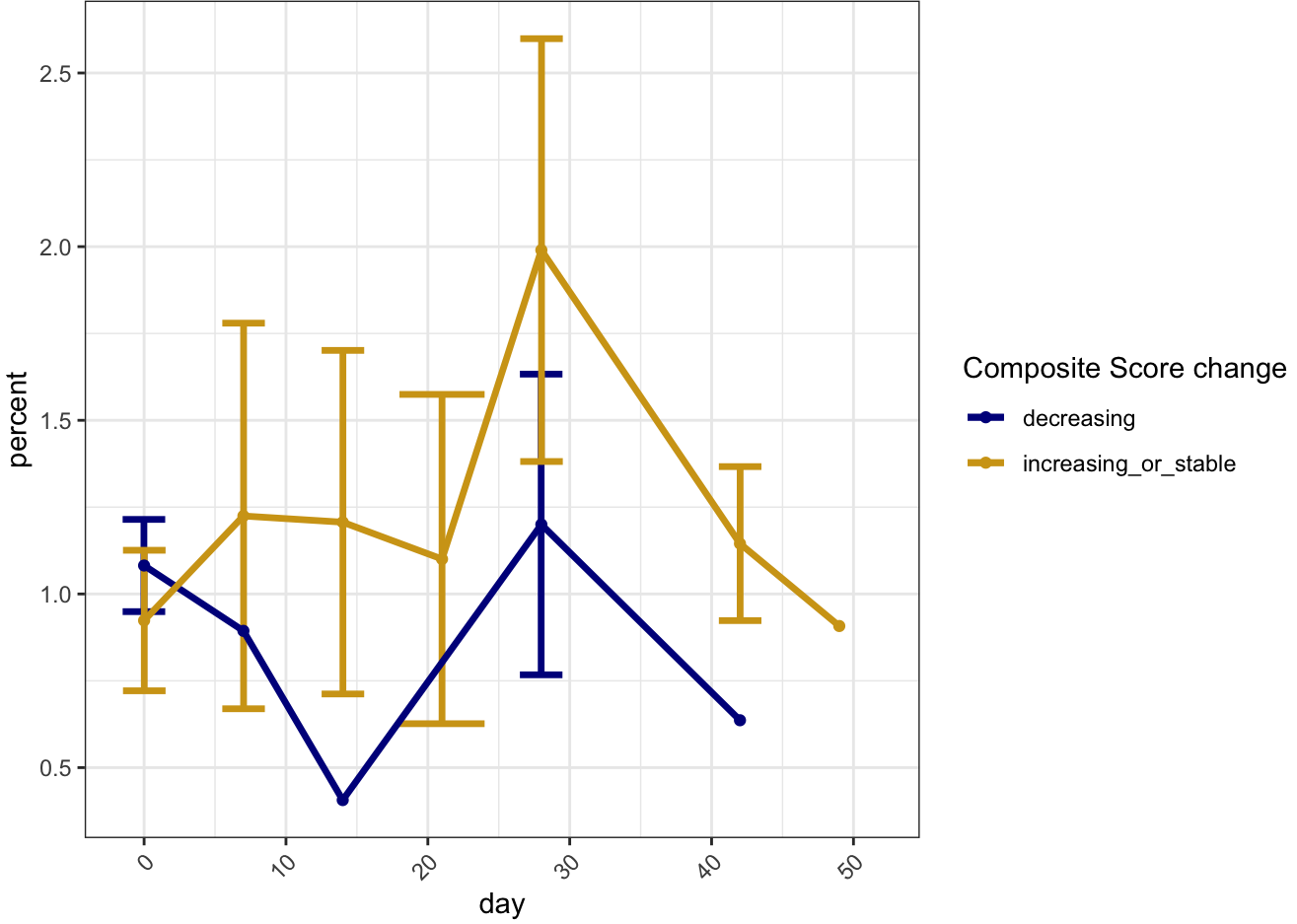


B)


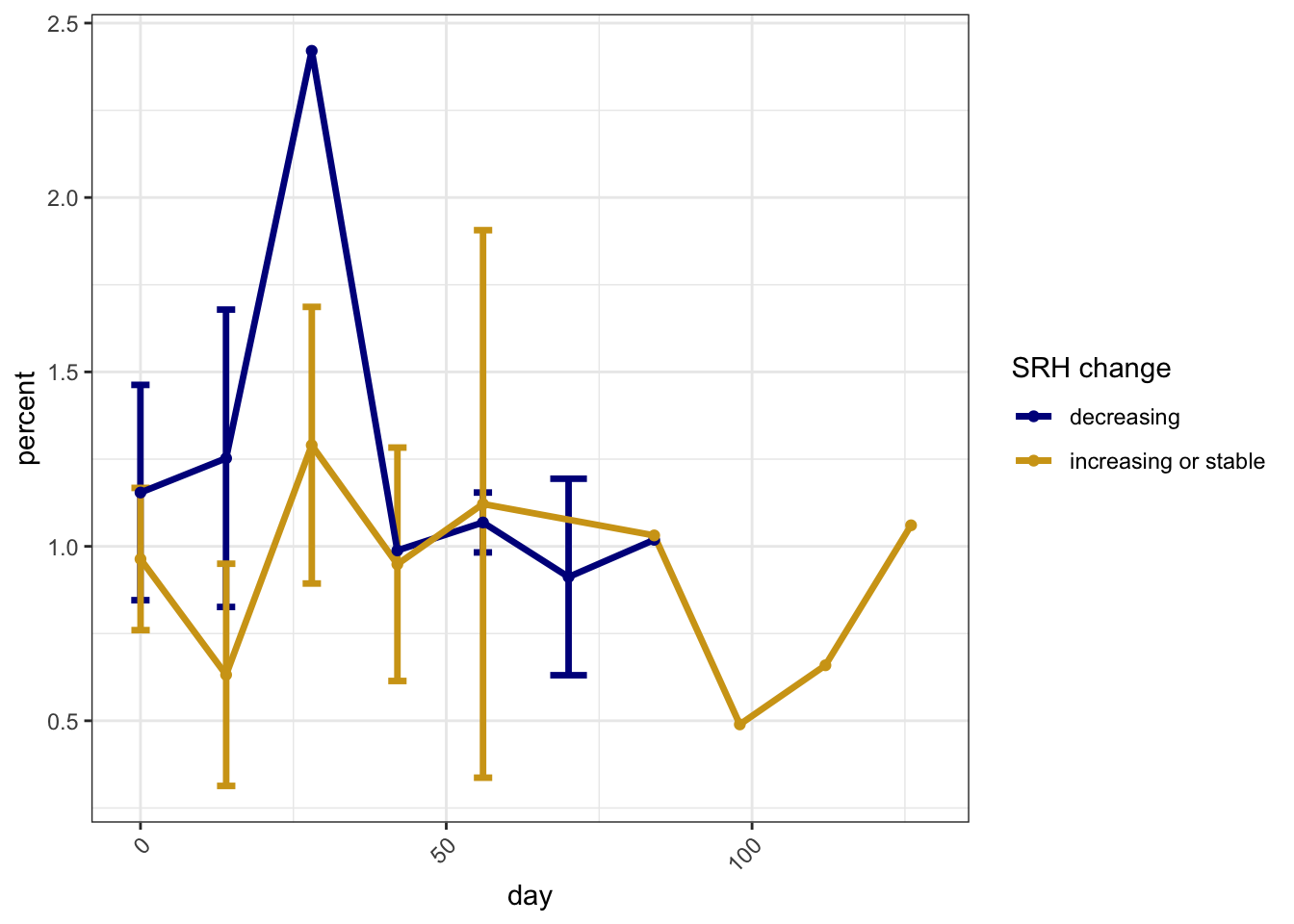


*Description: Exploratory line plots showing Activity Index (AInd) percentage change in relation to patient-reported outcomes. (A) Weekly percentage change in AInd stratified by composite symptom score slopes. Participants with stable or increasing symptom burden (gold) showed a modest upward trend, while those with decreasing scores (blue) showed an overall decline. (B) AInd percentage change in 14-day bins stratified by self-rated health (SRH) slopes.* *Duplicate same-day entries were collapsed by retaining the first response. No significant associations were observed. Error bars represent standard errors.*

**Table S1. Number of valid 24-h accelerometer Activity Index (AInd) intervals by self-rated health (SRH) change group and 7-day study bin**

| **Study day (start of 7-day bin)** | **Decreasing SRH (AInd n_valid_)** | **Increasing or stable SRH (AInd n_valid_)** | **Total (AInd n_valid_)** |
| --- | --- | --- | --- |
| 0 | 3 | 9 | 12 |
| 7 | 1 | 2 | 3 |
| 14 | 1 | 2 | 3 |
| 21 | 2 | 0 | 2 |
| 28 | 1 | 4 | 5 |
| 42 | 1 | 2 | 3 |
| 49 | 0 | 1 | 1 |
| 56 | 1 | 2 | 3 |
| 63 | 1 | 1 | 2 |
| 70 | 1 | 0 | 1 |
| 77 | 2 | 0 | 2 |
| 84 | 1 | 0 | 1 |
| 91 | 0 | 1 | 1 |
| 105 | 0 | 1 | 1 |
| 112 | 0 | 1 | 1 |
| 126 | 0 | 1 | 1 |
| **Total** | **15** | **27** | **42** |

Description: Counts represent 24-h accelerometer intervals used for AInd calculation. AInd was computed as the hourly mean of wearable accelerometer data during the daytime window (07:00–19:00). Intervals are summarized within 7-day study bins and stratified by overall SRH slope (decreasing vs increasing or stable). AInd required sufficient accelerometer data during the daytime window (07:00–19:00) to compute the hourly summary and did not include a non-wear time threshold. Intervals spanning more than 48 hours contributed only the first 24-hour segment, and patient-initiated monitoring episodes could contribute zero, one, or more AInd intervals.

**Table S2. Comparison of Activity and Body Composition Measures by Key Covariates**

| **Covariate** | **Group** | **Total Step Count (steps; Mean ± SD)** | **Cadence (steps/min; Mean ± SD)** | **LWB (steps; Mean ± SD)** | **Activity Index (arbitrary units; Mean ± SD)** | **Impedance (Ohms or Ω; Mean ± SD)** |
| --- | --- | --- | --- | --- | --- | --- |
| Overall | — | 8,685.2 (5,753.5) | 40.5 (18.2) | 1,541.0 (977.52) | 96,239 (39,098) | 391 (91.2) |
| >3 Prescriptions /day | No (n=3) | 12,357.6 (6,074.7) | 43.9 (9.0) | 2,026.8 (1,018.8) | 120,811 (35,605) | 391 (83.9) |
|  | Yes (n=6) | 5,502.4 (2,972.2)*** | 37.5 (23.4) | 1,119.9 (737.6)*** | 83,953 (35,231)*** | 391 (95.3) |
|  | Follow Up worse (n=1) vs not (n=8) | 5718.0 (-) vs 8913.4 (5912.4) | 46.5 (-) vs 40.0 (18.3) | 1455.6 (-) vs 2650.5 (1,218.3) | 80454.0 (-) vs 97,453.7 (40,096.9) | n/a |
| G8 score <14 | No (n=5) | 10,407.4 (6,215.5) | 42.9 (19.7) | 1,573.3 (1,011.1) | 100,120 (42,612) | 419 (76.3) |
|  | Yes (n=4) | 5,049.3 (1,697.3)*** | 35.4 (14.0) | 1,472.7 (957.6) | 91,976 (35,429) | 338 (94.6) |
|  | Follow Up worse (n=3) vs not (n=6) | 3,801.4 (1,628.4) vs 9,746.9 (5,791.8) *** | 25.3 (10.3) vs 43.8 (17.9)*** | 946.6 (830.77) vs 1,670.2 (974.41) | 84,240 (14,771) vs  99,989 (43,559) | 462 (49.8) vs 371 (90.2) |
| CARG >5 | No (n=5) | 11,074.6 (6,098.5) | 43.0 (9.5) | 1,955.1 (954.4) | 106,300 (38,319) | 404 (73.4) |
|  | Yes (n=4) | 5,499.3 (3,340.6)*** | 37.1 (25.8) | 988.8 (721.8)*** | 81,451 (36,390)*** | 378 (105) |
|  | Follow Up worse (n=3) vs not (n=6) | 9,746.3 (8,013.8) vs 7,998.6 (3,800.9) | 34.1 (12.9) vs 44.6 (20.2) | 1,585.6 (1,060.9) vs 1,512.1 (952.27) | 108,957.2 (44,309.8) vs 88,413.0 (34,084.0)** | 457 (51.8) vs 359 (89.3) |
| CIRS-G score > Median | No (n=4) | 11,404.3 (6,163.1) | 43.0 (9.9) | 1,927.5 (981.3) | 111,880 (39,956) | 401 (79.4) |
|  | Yes (n=5) | 5,547.7 (3,203.2)*** | 37.6 (24.7) | 1,094.9 (790.0)*** | 82,021 (33,095)*** | 383 (98.9) |
|  | Follow Up worse (n=2) vs not (n=7) | 4,052.5 (1,616.8) vs 9,948.6 (5,842.4)*** | 29.3 (16.5) vs 43.5 (17.7)** | 1,277.5 (1,214.2) vs 1,612.8 (923.2) | 91,976 (35,429) vs 100,120 (42,612) | 486 (45.3) vs 375 (87.2) |
| FACT-G score > Median | No (n=5) | 5,917.5 (3,152.2) | 42.6 (27.0) | 1,374.1 (1,033.5) | 75,747 (32,371) | 414 (82.9) |
|  | Yes (n=4) | 10,476.0 (6,401.3)*** | 39.1 (9.8) | 1,648.9 (955.7) | 113,168 (36,459)*** | 374 (93.9) |
|  | Follow Up worse (n=5) vs not(n=4) | 5,049.3 (1,697.3) vs 10,407.4 (6,215.5)*** | 35.4 (14) vs 42.9 (19.7) | 1,472.7 (957.56) vs 1,573.3 (1,011.1) | 91,976 (35,429) vs 100,120 (42,612) | 332 (86.5) vs 442 (59.1) |
| Health Status as good or better | No (n=2) | 8,359.6 (2,967.9) | 62.5 (27.5) | 2,037.4 (1195.3) | 58,797 (25,279) | 346 (64.3) |
|  | Yes (n=7) | 8,756.0 (6,244.6) | 35.7 (11.5)*** | 1,433.0 (919.1) | 105,050 (36,658)*** | 401 (93.7) |
|  | Follow Up worse (n=4) vs not (n=5) | 7,382.8 (4,476) vs 9,661.9 (6,520.6) | 34.6 (13.1) vs 44.8 (20.5) | 1,121.8 (755.3) vs 1,855.3 (1,027.5)*** | 96,726 (29,233) vs 95,909 (45,182) | 423 (86.1) vs 363 (86.9) |

**Abbreviations**: QOL, quality of life; CARG, Cancer Aging Research Group score; CIRS-G, Cumulative Illness Rating Scale-Geriatric.

* p < 0.2;
** p < 0.1;
*** p < 0.05

For groups with n=1 participant, SD and p-values are not reported

Description *Accelerometry‑derived activity metrics—total steps/day, cadence (steps/min; 90th‑percentile “best pace”), longest walking bout (LWB) (steps in the longest uninterrupted bout, capped at <2 hours), and the Activity Index (AInd) (device‑specific movement‑variability metric)—alongside bioimpedance (Ω), stratified by baseline geriatric assessment domains (polypharmacy >3 prescriptions/day; G8 <14; CARG >5; CIRS‑G > median; FACT‑G > median; SRH good/very good/excellent). “Worse vs not” rows display week‑12 change vs baseline within each GA domain using prespecified thresholds (More prescriptions, G8 decline ≥1 point; CARG increase ≥1 point; CIRS‑G increase ≥1 point; FACT‑G decline >3 points; any SRH downgrade). Higher steps, cadence, LWB, and AInd indicate greater mobility; AInd values are reported in arbitrary units and are not directly comparable to external thresholds but are internally consistent for between‑group comparisons.* *For AInd, we did not apply the 1.5-hour non-wear exclusion used for step-derived metrics, as AInd is variance-based and computed from available daytime epochs*
